# Medical Ethics and Informed Consent to Treatment: Past, Present and Future

**DOI:** 10.7759/cureus.75377

**Published:** 2024-12-09

**Authors:** Alan Mordue, Elizabeth A Evans, James T Royle, Clare Craig

**Affiliations:** 1 Public Health, Health Advisory and Recovery Team, London, GBR; 2 Health Policy, Health Advisory and Recovery Team, London, GBR; 3 Colorectal Surgery, Health Advisory and Recovery Team, London, GBR; 4 Pathology, Health Advisory and Recovery Team, London, GBR

**Keywords:** benefits and risks, covid-19 pandemic, informed consent, medical education and training, medical ethics

## Abstract

It has been asserted that there was an erosion of medical ethics during the Covid-19 pandemic and a departure from the principle of obtaining fully informed consent from patients before treatment. In light of these assertions, this article reviews the historical development of medical ethics and the approach to obtaining informed consent and critiques the consent practices before and during the pandemic. It then describes a new tool for displaying key statistics on the benefits and risks of interventions to help explain them to patients and suggests a more rigorous process for seeking fully informed consent in the future.

## Introduction

During the Covid-19 pandemic, it became increasingly apparent to some medical professionals that we were experiencing a global crisis in medical ethics [1]. They argued that we witnessed a dangerous erosion of the core ethical principles that underpin civilised society through the implementation of misguided and unethical Covid-19 policies by governments and public health bodies [2]. Well-established medical and public health principles were disregarded, such as the requirement for robust evidence, adhering to the precautionary principle in order to minimise risk, careful consideration of harms and benefits, fully informed consent to treatment and adherence to due legal process and the provision of accurate information to the public in a way that avoids undue alarm.

Certainly, many policies implemented during the pandemic period would have been completely unacceptable according to pre-pandemic norms of public health ethics and, arguably, have led to medicine being practised in a way that is dysfunctional and more dangerous for patients. As we take stock of the profound impact that these emergency policies have had on the practice of medicine, we contend that the medical profession must now take action to reclaim and restore the fundamental principles of patient-centred, ethical medicine.

Much of this article focuses on one of these principles, namely, informed consent, and at the individual patient and clinician level. However, the same principle applies to public health interventions whether (i) interventions are applied to whole populations with no ability to opt out (such as seatbelt legislation or lockdown closures of schools and businesses), (ii) interventions are recommended for sections of the population and where there is an ability to opt out (such as screening programmes or mask wearing during the Covid-19 pandemic) or (iii) individual interventions are applied to manage communicable diseases (such as advice on isolation and chemoprophylaxis with meningococcal disease or advice on isolation after Covid-19). In all situations, there should be a rigorous assessment of the evidence on benefits and risks, and decision-makers should be fully informed, whether they be parliamentarians on behalf of their constituents as in (i); national screening or vaccination committees and health ministers, for example, as in (ii); or individual patients as in (ii) and (iii).

Here, we present an overview of the development of medical ethical principles and specifically informed consent practices and how they were applied before and during the years of the Covid-19 pandemic, and finally, we propose a new tool to promote patient communication and suggest improvements to informed consent processes. An earlier version of this article was posted to the Open Science Framework (OSF) preprint server on April 23, 2024.

## Technical report

Past: Developing medical ethics and informed consent practice

The need for ethical principles to underpin the practice of medicine in order to protect patients from harm has been recognised for millennia, since Hippocrates, the Greek philosopher and physician known as the ‘father of modern medicine’, who is credited with establishing the Hippocratic Oath in around 400 BC [3]. Ethical codes and laws recognise the fundamental imbalance of power in a doctor-patient relationship, in which the doctor will always hold a privileged position of power and responsibility over the vulnerable patient. The establishment of ethical codes, laws and principles aims to protect the patient from potential abuse that could arise from this skewed power dynamic.

The Hippocratic Oath contains principles that give moral guidance and accountability to doctors, encompassing two key tenets: the doctor is required to act with beneficence (to help and prevent harm) and non-maleficence (to do no harm or ‘Primum non nocere’). These principles help to ensure that all medical treatment is necessary and proportionate so that the benefits of treatment outweigh the risks to the individual patient. Over time, two other principles have become accepted, namely, autonomy and justice, and together with beneficence and non-maleficence constitute the four pillars of medical ethics. Autonomy derives from the philosophies of Kant and Mill, relating to the intrinsic value of each individual and therefore their right to self-determination. Justice concerns the fair, equitable and appropriate treatment of individuals [4].

Informed consent, truth-telling and confidentiality in turn are derived from the principle of autonomy. Informed consent, for medical interventions or research, requires that the patient (i) must be competent to understand and decide, (ii) receives a full disclosure, (iii) comprehends the disclosure, (iv) acts voluntarily and (v) consents to the proposed action [4]. An autonomous patient has the right to know their diagnosis, prognosis and the effects of any treatment options (disclosure), and this truth-telling is essential for a trusting relationship with the doctor. In a similar fashion, the confidentiality of the patient’s medical and personal information is necessary to maintain and promote trust.

These principles of medical ethics have been developed and updated over the centuries and are enshrined today in national and international laws and codes. The Nuremberg Code (1947), although not legally binding, was a landmark document in medical and research ethics [5]. It was drawn up at the end of the Nuremberg trial of doctors, who had carried out medical experimentation on vulnerable prisoners during the Nazi regime. It outlawed involuntary experimentation on human subjects, emphasising that ‘…the voluntary consent of the human subject is absolutely essential…without the intervention of any element of force, fraud, deceit, duress, overreaching, or other ulterior form of constraint or coercion; and (the subject) should have sufficient knowledge and comprehension of the elements of the subject matter involved as to enable him to make an understanding and enlightened decision’.

A more modern statement of ethical principles for physicians undertaking medical research involving human subjects is the World Medical Association’s Declaration of Helsinki, which was first adopted in 1964 and most recently amended in October 2013 [6]. Both the Nuremberg Code and the more recent Declaration of Helsinki state that experimental treatment must only be carried out with the fully informed consent of the study participant.

Bodily autonomy is the foundation on which all other human rights are built and is enshrined in Article 3 of the European Union (EU) Charter of Human Rights, ‘Right to Integrity of the Person’ [7], and Article 6 of the United Nations Educational, Scientific and Cultural Organization (UNESCO) Universal Declaration on Bioethics and Human Rights (2005) [8], which states that ‘Any preventive, diagnostic and therapeutic medical intervention is only to be carried out with the prior, free and informed consent of the person concerned, based on adequate information’.

Doctors and other medical practitioners are required by their professional codes of practice to obtain informed consent for all medical treatments and interventions, following strict ethical criteria. For consent to be valid, a doctor must disclose to the patient all material risks (both known and unknown), as well as the benefits of the intervention and alternatives to treatment, including doing nothing. Consent must be completely voluntary, with no coercion or pressure applied by either the medical practitioner, family or the wider public, and the patient must have the capacity to consent. This so-called patient-centred care reflects such professional codes and is the gold standard of an ethical doctor-patient relationship, one where the doctors’ primary duty of care is to the individual patient in front of them, always acting in their best interest and considering their physical, emotional and philosophical or spiritual needs and wishes at all times.

The importance of fully informed consent and patient-centred decision-making is recognised in UK case law and strengthened in the UK Supreme Court Montgomery v Lanarkshire Health Board (2015) judgement, which stated the following [9]: ‘An adult person of sound mind is entitled to decide which, if any, of the available forms of treatment to undergo, and her consent must be obtained before treatment interfering with her bodily integrity is undertaken. The doctor is therefore under a duty to take reasonable care to ensure that the patient is aware of any material risks involved in any recommended treatment, and of any reasonable alternative or variant treatments…. The test of materiality is whether, in the circumstances of the particular case, a reasonable person in the patient’s position would be likely to attach significance to the risk’.

Furthermore, the judgement in Thefaut v Johnston (2017) EWHC 497 (QB) emphasised the duty of a doctor to give patients both accurate information and adequate time and space to make decisions about medical interventions [10].

Present: Informed consent before and during the Covid-19 pandemic

Over the latter part of the 20th century and the start of the 21st century, the traditional, paternalistic, ‘doctor knows best’ approach to medicine had gradually given way to a more patient-centred approach. The development of a social culture that was less deferential to doctors, along with easy access to information through the internet, had also created better-informed patients.

However, there appears to have been significantly more interest in improving the process of informed consent for surgical or invasive interventional procedures than for pharmacological therapeutics, despite the legal developments and requirements outlined in the previous section. This is likely multifactorial but may be particularly related to (i) the invasive and irreversible nature of surgical procedures, (ii) the need for the patient to place complete trust in the surgeon and anaesthetist, (iii) increased performance anxiety in surgeons as patients have become more independently informed and questioning, (iv) the increased technical challenge of some procedures, (v) concerns about operating on ‘high risk’ patients and finally (vi) the risk of litigation after complications. All these doctor- and patient-related factors have driven the improvements seen in the process of informed consent for surgery.

Obtaining informed consent for an elective surgical procedure is considered to be a process that occurs over a period of time and several conversations, beginning with an initial conversation in which the diagnosis and potential treatment options are explained to the patient. The risks and benefits of the various treatment options that the surgeon considers to be feasible and appropriate, including no treatment, are all discussed as valid options for the patient to consider. The patient’s general condition, comorbidities, functional status, lifestyle, expectations, hopes and aspirations must all be taken into account.

The patient should be given an information leaflet to take away detailing the nature of the procedure(s) proposed, what can be expected afterwards, recovery time, advice and guidance on possible restrictions, how to prepare in advance to optimise chances of success and tips to enhance post-operative recovery. The written information must include all the general and specific risks in material terms; any complication that is considered a material risk to that individual patient should be stated and quantified (after the Montgomery ruling), however rare. The patient may be given the consent form at this point but is not required to sign it, though the clinician might sign it. The patient should then be given adequate time (days or weeks) to consider their options before making a final decision and attending for the proposed procedure.

The consent form is usually signed by the patient on the day of the procedure or, if previously signed, countersigned again to confirm. A consent form is not a legally binding document, or a contract, although it may be used later by legal representatives in support of either the patient or the clinician. It is simply a document signed to indicate that an appropriate process of discussion, consideration and agreement as outlined above has taken place between the patient and the clinician.

The process described above for seeking informed consent for elective surgery is the gold standard; however, it is widely accepted that standards remain variable and do not always meet the ideal. A study by Courtney and Royle showed that even for a procedure as common as inguinal hernia repair, there was wide variation in the content of premade consent forms at different NHS hospitals [11]. The same authors reported that despite the fact that procedure-specific consent forms (PSCFs) have been shown to improve consenting practice, too few trusts are using PSCFs, and the listed risks and incidences on each PSCF are highly variable [12].

Despite the clear mandate for full and informed consent to be obtained by the doctor prior to all medical interventions, it is notable that there remains a significant difference in the process of consent that is followed between surgical procedures and the prescribing of pharmaceutical drugs or vaccines by doctors, especially in terms of the disclosure of all material risks to the individual and the time allowed for a decision to be made. We argue that the same level of rigour of risk disclosure and weighing up risks against benefits for the individual should be applied to all interventions, whether surgical or pharmaceutical. For elective surgery and ‘elective’ long-term medications, a rigorous process should be followed, whereas for emergency and acute, short-term medications, a shorter and simpler process could be followed, as for emergency surgery. In addition, when it comes to preventative vaccines given to healthy populations (as opposed to drugs prescribed to treat a sick person), individuals may have a far lower ‘risk appetite’ for adverse outcomes and side effects, requiring a much lower risk profile to be acceptable. Furthermore, although many adverse reactions from drugs are reversible by stopping the drug (although this is clearly not possible with a vaccine), that is not always the case, and consent for the risk of irreversible harm should be given more weight.

In the immediate pre-pandemic period, this was the situation in medical and surgical practice. So, what happened during the pandemic itself, specifically in relation to the main medical intervention deployed, the Covid-19 ‘vaccines’?

From January 2021, (i) there was a rapid rollout of the novel mRNA and DNA vaccine technology under emergency authorisation and with no long-term safety data to almost the whole population, most notably children and pregnant women, regardless of their individual risk from Covid-19 or their immune status with respect to SARS-CoV-2 (compromises the principles of truth-telling/full disclosure, beneficence and non-maleficence) [13]. A recent paper performed a risk-benefit assessment and ethical analysis and concluded that ‘booster mandates in young adults are expected to cause a net harm’ [14]. Next, (ii) vaccine benefits were exaggerated, going beyond the reduction of symptomatic cases observed in the trials [15], with many in positions of authority claiming that mass vaccination would stop Covid-19 transmission through vaccine-induced herd immunity [16] and some stating that vaccination was the only route to avoiding ongoing lockdowns and masking (compromises truth-telling/disclosure and voluntary consent); (iii) governments and health officials heavily marketed the Covid-19 vaccines to the public using terms such as ‘safe and effective’ and ‘rigorously evaluated by MHRA’ (UK Medicines and Healthcare products Regulatory Agency) making an individual objective risk versus benefit analysis in order to give valid informed consent more difficult (compromises truth-telling/disclosure and voluntary consent); (iv) the requirement to disclose alternatives to the Covid-19 vaccines was not followed: information concerning potentially effective drugs for early treatment was not shared with the public [17], leaving those at significant risk with no apparent choice (compromises truth-telling, beneficence and non-maleficence); (v) in many countries, Covid-19 vaccines were mandated for certain groups and in order to access certain basic human freedoms (e.g. work, travel, shopping and leisure), a violation of the principle that consent must be entirely voluntary and without coercion, penalty or restriction (compromises autonomy and voluntary consent).

Next, (vi) there was a failure of regulators and governments around the world to interrogate or respond to safety signals in vaccine safety surveillance systems over the last three years (compromises truth-telling and non-maleficence) [18], and furthermore, (vii) when concerned professionals and vaccine-injured patients did try to inform people of the risks, they were smeared and silenced (compromises truth-telling/disclosure) [19]. Finally, the actions taken by governments to indemnify the Covid-19 vaccine manufacturers against liability for harms suffered, and the often very limited and inadequate government compensation schemes, have left patients holding the full risk.

It is hard not to conclude that the medical profession has stood by, largely silent, as under emergency laws in the United Kingdom and around the world, one by one, fundamental ethical principles have been abandoned and previously hard ethical red lines have been crossed. In Europe, this occurred despite the Parliamentary Assembly of the Council of Europe passing Resolution number 2361 on 27 January 2021, which states the following [20]: 1.1 Paragraph 7.3.1 - ‘ensure that citizens are informed that (the Covid-19) vaccination is NOT mandatory and that no one is politically, socially, or otherwise pressured to get themselves vaccinated, if they do not wish to do so themselves’; 1.2 Paragraph 7.3.2 - ‘ensure that no one is discriminated against for not having been vaccinated, due to possible health risks or not wanting to be vaccinated’.

In challenging times and emergencies, it is particularly important that the medical profession and wider society continue to respect and uphold ethical principles, not just when things are going well: Their primary purpose is to safeguard patients and to prevent abuse and atrocities, and this is particularly pertinent in an emergency situation, as the risk of abuse is far higher when panic and fear are present and may influence decision-making.

Future: Improving information for patients and consent practices

We contend that it is imperative for the medical profession to implement a more rigorous process of informed consent for pharmaceutical interventions, including vaccines, to bring the standard to the level of consent for surgical procedures. In 2015, Malhotra et al. described a US initiative to help doctors stop using interventions with no benefit [21]. They observed that ‘A culture of “more is better,” where the onus is on doctors to “do something” at each consultation has bred unbalanced decision making. This has resulted in patients sometimes being offered treatments that have only minor benefit and minimal evidence despite the potential for substantial harm and expense. This culture threatens the sustainability of high quality healthcare and stems from defensive medicine, patient pressures, biased reporting in medical journals, commercial conflicts of interest, and a lack of understanding of health statistics and risk’.

In their paper, they called for tools to be developed ‘that help clinicians to understand and share decisions on the basis of best evidence. Rather than prespecifying the outcome of such dialogue, and trying to get medicine “just right,” they should try to ensure that decisions are based on the best match between what is known about the benefits and harms of each intervention and the goals and preferences of each patient’. Below, we describe such a tool that could have great practical benefits to help doctors share the risks and benefits in an easy-to-understand way with their patients.

If patients are indeed to make truly informed decisions on their treatment, then what data will they need? The earlier consideration of ethical principles and in particular the need for truth-telling and disclosure point to four key areas: (i) risks from the disease in question (prognosis), (ii) the degree of benefit from the recommended treatment and any alternatives (beneficence), (iii) the risks of side effects and those for any alternatives (non-maleficence) and finally (iv) any significant uncertainties in any of these areas (truth-telling/full disclosure).

Taking the first of these key areas, an individual patient’s risk from a disease is a product of disease incidence (how likely they are to contract the disease) and, if they do, the likelihood of severe outcomes including death.

The benefit from a treatment, ideally assessed through randomised double-blind placebo-controlled trials, is usually described in terms of relative and absolute risk reduction (RRR and ARR). Pharmaceutical companies seem to prefer the use of RRR since the numbers are larger and, therefore, portray their drug in a more positive light than ARR. While both are useful, arguably, the ARR is less likely to mislead and gives a better estimate of the real-world impact and an easier comparison to side effect rates, which are usually expressed in absolute risk terms.

For example, consider a new treatment that randomised controlled trials (RCTs) have shown reduces the risk of catching a disease. If the disease is common, say 10 in 1,000 people may be affected, and the new treatment reduces the risk to six in 1,000, then the ARR is simply 10 minus six or four in 1,000 (see middle column in Table 1).

**Table 1:** Absolute and relative risk reduction compared

Risks	Common Disease/Outcome	Rare Disease/Outcome
Original risk	10 in 1,000	10 in 1,000,000
Risk after treatment	6 in 1,000	6 in 1,000,000
Absolute risk reduction	4 in 1,000	4 in 1,000,000
Relative risk reduction	4/10 x 100 = 40%	4/10 x 100 = 40%

The reduction in risk of 4 can be related to the original risk of 10 to calculate the RRR, usually expressed in percentage terms by multiplying by 100, so 40%. However, if the disease is rare, for example, 10 in a million, although the RRR is exactly the same at 40%, the ARR is very different, four per 1,000,000 (right-hand column). A patient may take a very different view about the degree of benefit; four per 1,000 or four per million, especially if the treatment is inconvenient to take, is likely to give them immediate side effects or carries a longer-term risk of serious side effects.

The charts below present an example for a common, real-world example of statins (taken to reduce the risk of cardiovascular disease {CVD}) and provide data for men and women separately on the first three key areas outlined above. We describe below how this approach is a useful tool in the informed consent process, helping the doctor to convey the risks and benefits of statins in a way that is individualised for the patient and in a format that hopefully improves understanding.

**Figure 1:**
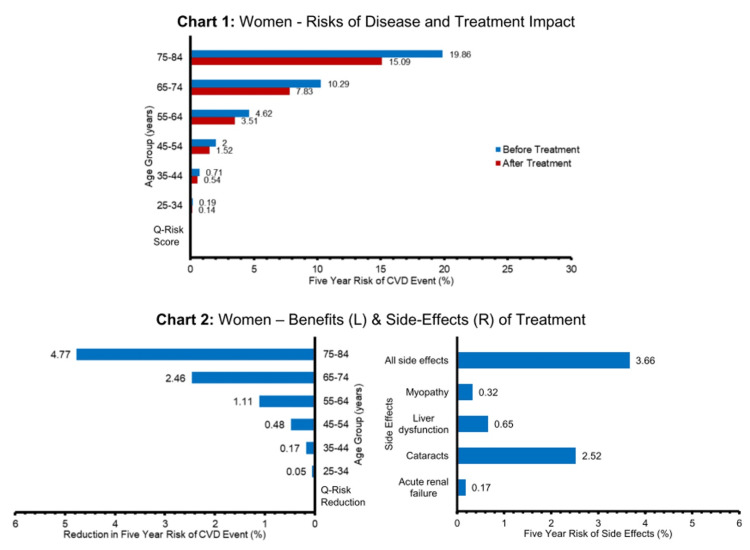
Cardiovascular risk and statin benefits and side effects in women CVD: cardiovascular disease

Using Charts 1 or 3 and based on their age and sex, a doctor can show the patient their approximate absolute risk of developing a cardiovascular disease (CVD) event (defined as fatal or non-fatal ischaemic stroke or coronary heart disease, therefore covering a range of severity from angina and transient ischaemic attacks to death) over the next five years (blue lines) (Figure 1) [22]. The doctor could also calculate a more specific risk for their patient using an online calculator such as QRISK3 and plot their individualised risk on the chart [23]. They can then show the patient the estimated risk they would achieve if they took statins daily for the next five years (red lines) [24]. By multiplying an individual QRISK3 score by 0.76, the lower risk on statins can be calculated (see Appendices for data sources and assumptions).

**Figure 2:**
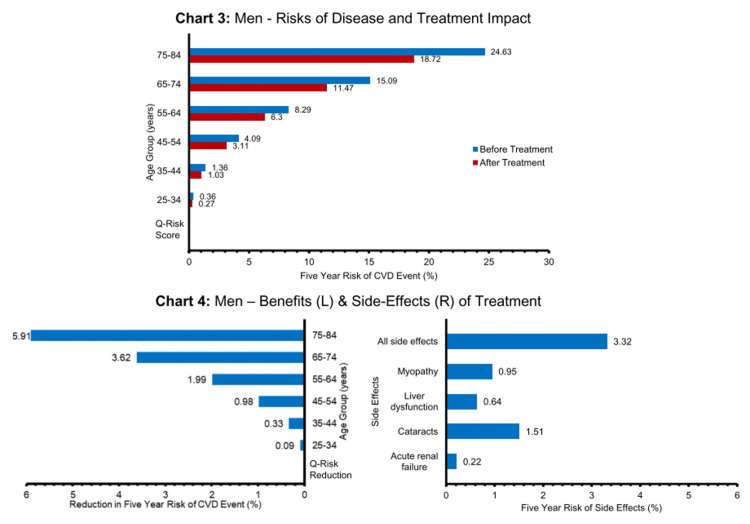
Cardiovascular risk and statin benefits and side effects in men CVD: cardiovascular disease

The left-hand side of Charts 2 and 4 shows the difference between the original absolute risk of CVD and the lowered risk with statins, that is, the ARR or treatment benefit (Figure 2). So next, the doctor can discuss the approximate ARR, based on the patient’s age and sex or the individualised QRISK3 score ARR. The right-hand side of Charts 2 and 4 enables the doctor and patient to compare the risk of significant side effects from the drug over the next five years with their ARR over the same time period, providing a direct comparison of the benefit from treatment to the risk of serious adverse events (both for single side effects and all together) [25].

It is clear from this direct comparison of benefits to the risk of side effects that a 50-year-old woman might take a different view about the merits of taking statins than a 70-year-old man.

The fourth key area, that is, any remaining significant uncertainties, can be related to a disease, the benefits of treatment and the side effects (Table 2).

**Table 2:** Remaining areas of uncertainty

Area	Specifics
Disease risk	Disease incidence, severity or mortality
Chance of benefits	Impact on disease incidence (cases), severity or mortality. Evidence of benefit in particular groups, for example, children and pregnant women
Risk of side effects	Short- and long-term side effects, including evidence on fertility, teratogenesis and carcinogenesis

To facilitate patient understanding and the use of this ‘tool’ or framework, clear instructions and guidance to explain the charts and the remaining areas of uncertainty would be required. This guidance could include web links to up-to-date charts, needed, for example, if disease incidence is changing, if mortality rates are changing or if new side effects have emerged. These web links could also provide real-world comparison risks for other outcomes to give perspective, for example, the annual risk of serious injury/death as a result of a road traffic accident.

The data and remaining uncertainties presented in this informed consent tool would need to be agreed upon at the time of a new drug’s approval, with periodic updates as necessary. This agreement would, therefore, necessitate the involvement of regulators such as the MHRA, as part of the drug approval process. Critically, it is essential that the body charged with collating and deciding on the data for this tool is independent of both the pharmaceutical industry and the government.

A tool such as this to summarise and present key information for patients should be helpful but needs to be combined with a clear process to engage and support patients to reach their decision on treatment and consent. This process should include the following key principles and stages: firstly, verbal and written information displaying key data plus web links, such as in the tool presented here; secondly, honesty from the doctor about what is known and what is not, outlining remaining areas of uncertainty as in Table 2; thirdly, adequate time for patient consideration and discussion with others; and fourthly, a second appointment for further questions and clarifications.

Throughout, the role of the doctor is to provide information and to support the patient to reach a decision they are comfortable with. At the same time, we must recognise that not all patients may wish to engage fully with this process: Some may prefer to simply accept their doctor’s recommendation, and this preference should be respected. At all times, the doctor should act as the patient’s advocate, fulfilling their primary duty of care, which is to their patient, not influenced by wider considerations.

## Discussion

There is an urgent need for a doctor-driven return to ethical, patient-centred care, which is safeguarded against the vested interests of the pharmaceutical industry and inappropriate political influence. This was eloquently articulated in a paper in January 2023, which analysed the UK Health Security Agency (UKHSA) data indicating that the Covid-19 vaccines may have paradoxically increased, rather than decreased, overall mortality rates [26]. The authors concluded the following: ‘The modern medical system of many westernized countries has forsaken its ethical center, which is best summarized by the ancient Latin dictum of Primum Non Nocere, “First, Do No Harm.” The only way for medical doctors to reclaim the ethos of their once noble profession, and to do so with grace and respect, is to stop acquiescing to corrupt federal agencies and pharmaceutical influence, to take medicine back into their own hands’.

The concept of ‘evidence-based medicine’ (EBM), introduced in the 1990s, undoubtedly focused attention on the better appraisal, use and generation of evidence with the laudable aim of better patient care and outcomes. However, over time, it has arguably become increasingly reductionist and highly selective in what is considered acceptable evidence, with a danger of being dominated by pharmaceutical company-generated evidence. For example, in the context of Covid-19, we had several large and expensive RCTs in relation to potential new vaccines and new antiviral drugs but none in relation to vitamin D or ivermectin, generic products with no potential for large profits. This has allowed EBM to be hijacked by powerful vested interests who are able to use it to create a market for their drugs and vaccines, influencing medical practice by individual doctors for financial gain. There is now clear evidence of regulatory [27], scientific, academic [28] and political capture by the pharmaceutical industry, benefitting commercial interests over patients.

Moreover, key organisations involved in EBM, such as the National Institute for Health and Care Excellence (NICE), have been granted excessive power over the last couple of decades, with the increasing adoption of protocol-driven (technocratic) medicine over patient-centred (Hippocratic) medicine. Evidence-based guidelines have gradually come to be viewed as strict protocols and led to a ‘one size fits all’ approach to medicine: this undermines the informed consent process and the flexibility of doctors to support patients to make their own individual decisions on treatment. This dehumanises patients and deprofessionalises and deskills doctors, potentially leading to avoidable and unnecessary harm. It has also allowed organisations to effectively practise medicine on individuals, through protocols implemented from the top-down. This, combined with the rising acceptance of a ‘greater good’ political ideology, in which individual harm is accepted for the good of society, has created a medical environment that devalues the sanctity of each human life and makes patients highly vulnerable to iatrogenic harm and abuse.

It has always been understood that medicine is a fine balance of both art and science. Doctors are required to interpret science through an ethical framework, to enable medicine to be practised humanely, using both the head and the heart [4]. The overemphasis on the science of medicine and the resulting loss of the more humane and ethical art of medicine (relating to compassion, discernment, trustworthiness, integrity and conscientiousness) has damaged the relationship between doctors and patients and, arguably, led to worse outcomes for patients.

To reverse the tide, there are several steps that doctors can take: (i) relearn the art of medicine and treat every patient with dignity and respect as a doctor’s duty is primarily to the patient in front of them, to be their advocate and always act in their best interest; (ii) improve their practice of informed consent, particularly for long-term pharmaceuticals, by providing both verbal and easy-to-understand written information on benefits and side effects where possible tailored to the individual patient (such as the tool presented earlier), giving patients adequate time for reflection, questioning and decision, and being honest about remaining areas of uncertainty; (iii) understand that true science is never settled and embrace scientific debate, rejecting any ‘cancel culture’ or censorship in the medical sphere; and (iv) regain the habit and skills of critical thinking, reading and analysing key primary source studies and retaining a healthy scepticism when reading reviews and opinion pieces in the medical press. It is essential to avoid dogmatic positions and to have the intellectual humility to be able to change opinion when the evidence changes.

Furthermore, in relation to guidelines and protocols, doctors need to ensure the following: (v) There is a wide debate and transparency in their production and easy ways to critique them and provide feedback; (vi) there is a greater acceptance and support for clinicians who decide to deviate from them, where they believe doing so is in their patient’s best interest as guidelines can be very helpful but careful interpretation and judgement by clinicians are also required taking account of each patient’s unique circumstances; and moreover, (vii) there needs to be a broadening of the practice of medicine beyond the modern, narrow pharmaceutical model to include lifestyle changes to prevent disease and other more natural and traditional medicine approaches to help patients, particularly given their greater focus on supportive interactions.

Finally, (viii) doctors need to uphold agreed core ethical principles and re-establish ethical red lines, which should not be crossed, whatever the circumstances, and they must be alert to and call out corruption, fraud and vested interests.

Encouragingly, during the last three years, there have been several initiatives led by the medical profession to re-establish medical ethics. For example, the International Physicians’ Declaration (Rome, September 2021) sought to reclaim and invoke the Hippocratic Oath and its fundamental principles for humane, ethical medicine and condemned global governments’ Covid-19 policies [29]. This declaration has since been updated and expanded and signed by over 17,000 doctors worldwide. The UK Doctors Declaration (London, October 2022) was a public declaration signed by hundreds of UK doctors, stating ‘our grave concern about the violation of medical ethics that has become apparent in both clinical practice and scientific research, with the potential to result in serious harm to patients’ [30].

## Conclusions

Prior to the Covid-19 pandemic, the practice of informing patients and obtaining consent for treatment was far from ideal, but the responses to the pandemic laid bare just how far we had departed from acceptable standards of medical ethics. The post-pandemic period provides a great opportunity for the medical profession to learn from mistakes and comprehensively reject the move to technocratic medicine and put Hippocratic medical ethics back at the heart of the practice of medicine.

To do this, doctors must (i) accept that their primary duty is to their patient, always acting as their advocate and putting their interests first; (ii) improve their practice of informed consent by providing both verbal and written information on benefits and side effects, giving patients adequate time to reach a decision, and being honest about uncertainties; (iii) understand that science is never settled and embrace scientific debate, rejecting any censorship in medicine; (iv) regain the habit of critical thinking, reading key primary studies and retaining a healthy scepticism of reviews and opinion pieces; (v) engage in the debate about guidelines and accept the need to depart from them on occasion; (vi) broaden their practice beyond the narrow pharmaceutical model to include lifestyle changes to prevent disease and more traditional approaches with a greater focus on supportive interactions; and finally (vii) uphold core ethical principles and red lines and call out corruption, fraud and vested interests.

## Appendices

Sources and assumptions behind charts

Charts 1 and 3

Baseline risk: 10-year risk halved to derive five-year risk from Table 2 in the study of Hippisley-Cox et al. [22].

Treatment impact: from NICE Guideline 238 [24].

Table 3 shows key relative risk statistics from the guideline evidence review.

**Table 3:** Relative risks with statin treatment from NICE Guideline 238 CVD, cardiovascular disease; CV, cardiovascular; NICE, National Institute for Health and Care Excellence; MI, myocardial infarction

Outcome	Statin intensity		
	Low	Medium	High
All-cause mortality	0.89	0.85	0.91
CVD mortality	0.84	0.81	0.80
Non-fatal MI	0.78	0.62	0.51
Non-fatal ischaemic stroke	0.83	0.73	0.74
Major adverse CV event	0.85	0.91	0.71

Hippisley-Cox and Coupland quote an RR of 0.76 for major CVD events [25].

Assumed RR for CVD events = 0.76. Given the variation is above RRs (and the confidence intervals around them are not shown), charts could show ‘credible limits’; however, for simplicity, this has not been undertaken here.

Charts 2 and 4

Benefit of treatment (L): Reduction in five-year absolute risk of CVD events calculated by subtracting risk after treatment from that before treatment, for example, in Chart 3 for men aged 75-84 years: 24.63 - 18.72 = 5.91%.

Side effects of treatment (R): Taken from Table 7 in the study of Hippisley-Cox and Coupland [25] using ≥15% CVD risk (note: study covered 35-74 age range only).
